# Psychiatric comorbidity and pain catastrophizing in outpatients with chronic headache: a clinical series

**DOI:** 10.1192/j.eurpsy.2026.11484

**Published:** 2026-07-31

**Authors:** I. Martin Villalba

**Affiliations:** Psychiatry and Clinical Psycholoy, Hospital Clinic de Barcelona, Barcelona, Spain

## Abstract

**Introduction:**

Chronic headache, including migraine and tension-type headache, is a leading cause of disability worldwide and frequently leads patients to seek care in specialized outpatient clinics. Psychiatric comorbidity and maladaptive cognitive patterns such as pain catastrophizing contribute to headache chronification and poor treatment response. The Health Psychology Section plays a key role in evaluating these psychological factors in outpatient settings.

**Objectives:**

To describe the psychopathological profile, emotional symptoms, and pain catastrophizing in a clinical series of outpatients with chronic headache evaluated by the Health Psychology Section due to persistent symptoms despite medical treatment.

**Methods:**

A descriptive study of 10 outpatients (8 women, 2 men; aged 22–58) attending the headache specialty clinic at Hospital Clínic i Provincial de Barcelona. Patients were assessed by the Health Psychology Section with a clinical interview, the Hospital Anxiety and Depression Scale (HADS), and the Pain Catastrophizing Scale (PCS). Clinical data on headache duration, analgesic use, and psychiatric history were recorded.

**Results:**

Eighty percent of patients exhibited clinically relevant anxiety symptoms, and 60% had depressive symptoms. Four patients had a prior psychiatric diagnosis, mainly mood and anxiety disorders. High levels of pain catastrophizing were identified in 70% of patients, especially in rumination and helplessness domains. Patients reported high emotional distress and low perceived control over pain. Analgesic overuse was reported in 6 patients.

**Conclusions:**

Outpatients with chronic headache frequently present with psychiatric symptoms and maladaptive pain cognitions such as catastrophizing. Psychological assessment by the Health Psychology Section enables early identification of these factors, facilitating biopsychosocial interventions. Addressing both emotional distress and dysfunctional pain beliefs is essential to optimize treatment outcomes and reduce chronicity in this population.

**Disclosure of Interest:**

None Declared

